# The prognostic nutritional index as a predictor of cardiovascular and all-cause mortality in chronic kidney disease: a population-based analysis of NHANES data (1999–2018)

**DOI:** 10.3389/fmed.2025.1589173

**Published:** 2025-06-06

**Authors:** Weiwei Li, Lumiao Chen, Linsen Jiang, Zhijian Zhang, Kai Song

**Affiliations:** ^1^Department of Nephrology, The Second Affiliated Hospital of Soochow University, Suzhou, China; ^2^Department of Nephrology, The Affiliated Wuxi People’s Hospital of Nanjing Medical University, Wuxi People’s Hospital, Wuxi Medical Center, Nanjing Medical University, Wuxi, Jiangsu, China

**Keywords:** prognostic nutritional index, nutritional status, lymphocyte count, chronic kidney disease, cardiovascular mortality, all-cause mortality, mediation analysis

## Abstract

**Objective:**

This study evaluates the predictive value of prognostic nutritional index (PNI) for all-cause and cardiovascular mortality in chronic kidney disease (CKD) patients based on data from the National Health and Nutrition Examination Survey (NHANES), and to explore its variability across different CKD stages.

**Methods:**

A total of 4,528 CKD patients from the NHANES database (1999–2018) were included. Cox regression models were used to analyze the association between PNI quartiles (Q1–Q4) and mortality risk. Restricted cubic spline (RCS) analysis was employed to explore non-linear relationships, and subgroup and mediation analyses were conducted.

**Results:**

Patients in low PNI group (Q1) exhibited significant metabolic disturbances including elevated blood urea nitrogen and creatinine, reduced albumin and estimated glomerular filtration rate (eGFR). Compared to the Q4 group, the Q1 group had a 67% increased risk of all-cause mortality (HR: 0.598, 95% CI: 0.517–0.692) and a 103% increased risk of cardiovascular mortality (HR: 0.492, 95% CI: 0.374–0.648). RCS analysis revealed a non-linear relationship between PNI and mortality risk (threshold: 52), with significant predictive efficacy in CKD stages 1, 4, and 5 (*P* < 0.05), but not in stages 2 and 3 (*P* > 0.05). Mediation analysis indicated that age partially mediated the association between PNI and mortality (indirect effect proportion: 33%), while eGFR showed no mediating effect (*P* > 0.05).

**Conclusion:**

PNI is an independent predictor of all-cause and cardiovascular mortality in CKD patients. Future longitudinal studies are warranted to validate its clinical utility and intervention potential.

## 1 Introduction

Chronic kidney disease (CKD) has emerged as a global public health crisis, with its prevalence steadily rising, leading to significant health burdens due to cardiovascular complications and high mortality rates (1, 2). According to the World Health Organization, approximately 10% of adults worldwide are affected by CKD, with cardiovascular mortality rates 3–4 times higher than in the general population, escalating exponentially as renal function declines (3, 4). The latest reports showed that CKD patients with cardiovascular diseases (CVD) were associated with all-cause mortality and cardiovascular mortality (5, 6). Patients with comorbid cardiac and renal diseases exhibit higher mortality rates, with oxidative stress, inflammatory responses, and metabolic dysregulation collectively contributing to the underlying pathophysiology (7, 8). Although clinical guidelines emphasize the central role of nutritional management in CKD treatment, traditional assessment tools, such as body weight, BMI, serum albumin, fail to integrate immune status and metabolic disturbances, limiting their ability to accurately predict outcomes (9). Therefore, there is an urgent need for a biomarker that comprehensively reflects nutritional-immune homeostasis to optimize risk stratification in CKD patients.

The prognostic nutritional index (PNI), which combines serum albumin and peripheral lymphocyte count, offers dual value by assessing both nutritional status and immune function (10–12). Recent studies have demonstrated superior predictive performance of PNI for mortality risk in oncology and CVD (13, 14); however, its application in the CKD population remains underexplored. Given the prevalent microinflammation, immune dysregulation, and protein-energy wasting in CKD patients (9, 15), PNI theoretically provides a more comprehensive reflection of the pathophysiological features and their association with clinical outcomes. Nevertheless, existing studies have predominantly focused on single indicators or small cohorts (10), lacking large-scale population-based evidence to validate the independent predictive efficacy of PNI for cardiovascular and all-cause mortality in CKD patients, as well as its heterogeneity across different CKD stages.

This study, leveraging data from the National Health and Nutrition Examination Survey (NHANES, 1999–2018), is the first to systematically investigate the predictive value of PNI for cardiovascular and all-cause mortality risk in a large, representative CKD population. The findings aim to provide an economical and accessible biomarker for early risk stratification in CKD patients, guiding personalized nutritional support and immunomodulatory strategies to improve survival outcomes.

## 2 Materials and methods

### 2.1 Study population

**FIGURE 1:**
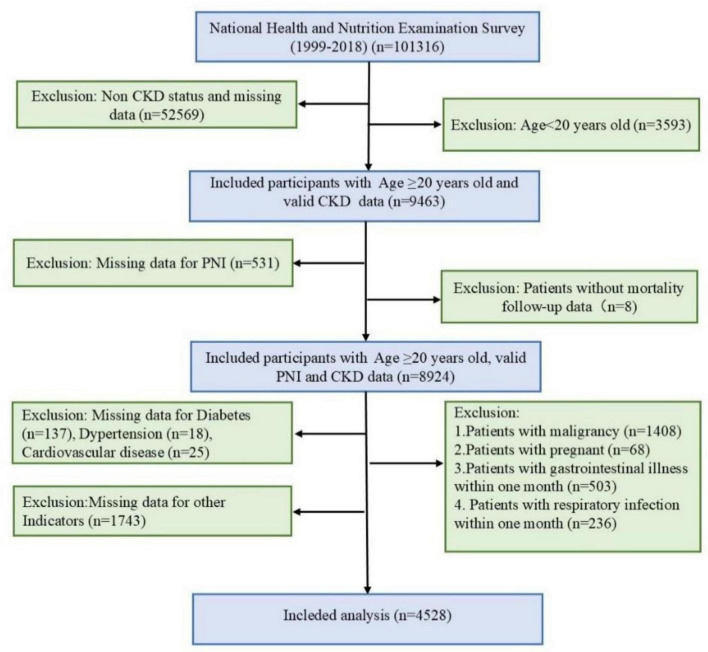
Screening flow of respondents.

This study utilized data from the NHANES conducted between 1999 and 2018. NHANES is a nationally representative cross-sectional study employing a multistage, stratified, random sampling design to collect health and nutritional information from the non-institutionalized US population. The inclusion criteria were as follows: (1) age ≥ 20 years; (2) completion of relevant questionnaires, physical examinations, and laboratory tests during the survey period; (3) availability of complete data for calculating the PNI; and (4) availability of complete diagnostic indicators for CKD and other covariates. To ensure the reliability and comparability of the analysis, exclusion criteria were applied: (1) individuals aged < 20 years; (2) patients with a history of malignancy; (3) pregnant women; (4) patients with acute respiratory or gastrointestinal infections within the past month; and (5) patients lacking mortality outcomes or event records during follow-up. The final study sample was representative of the overall characteristics of the US adult CKD population. A detailed flowchart is presented in Figure 1. Sampling weights and statistical analyses were adjusted to account for the complex sampling design of NHANES, ensuring the generalizability of the results.

### 2.2 Definition of CKD

CKD was defined according to internationally recognized standards, utilizing available data from the NHANES database. The diagnosis was based on the following two criteria: 1. estimated glomerular filtration rate (eGFR): eGFR was calculated using the CKD-EPI (Chronic Kidney Disease Epidemiology Collaboration) formula (16). For serum creatinine (Scr) ≤ 0.7 mg/dL (females) or ≤ 0.9 mg/dL (males), the formula was: eGFR = 144 × (Scr/0.7)∧−0.329 × (0.993)∧age × (1.159 if African American). For Scr > 0.7 mg/dL (females) or > 0.9 mg/dL (males), the formula was: eGFR = 144 × (Scr/0.7)∧−1.209 × (0.993)∧age × (1.159 if African American). Here, Scr represents serum creatinine (mg/dL), age is in years, and a factor of 1.159 is applied for African American participants. CKD was defined as eGFR < 60 mL/min/1.73 m^2^ sustained for at least 3 months. 2. Urine Albumin-to-Creatinine Ratio (UACR): UACR was calculated as the ratio of urine albumin concentration (mg/L) to urine creatinine concentration (g/L). Proteinuria was defined as UACR ≥ 30 mg/g. Participants meeting either of the above criteria were classified as CKD patients. Additionally, CKD stages were categorized according to the KDIGO (Kidney Disease: Improving Global Outcomes) guidelines (17): CKD Stage 1: eGFR ≥ 90 mL/min/1.73 m^2^ and UACR ≥ 30 mg/g; CKD Stage 2: eGFR 60–89 mL/min/1.73 m^2^ and UACR ≥ 30 mg/g; CKD Stage 3: eGFR 30–59 mL/min/1.73 m^2^; CKD Stage 4: eGFR 15–29 mL/min/1.73 m^2^; CKD Stage 5: eGFR < 15 mL/min/1.73 m^2^.

### 2.3 Measurement of PNI

The PNI was calculated using serum albumin levels and peripheral blood lymphocyte counts based on the following formula: PNI = 10 × serum albumin concentration (g/dL) + 0.005 × peripheral blood lymphocyte count (/μL) (18). Currently, there is no established classification standard for PNI. In this study, PNI values were categorized into quartiles: Q1: PNI < 49.0; Q2: 49.0 ≤ PNI < 52.5; Q3: 52.5 ≤ PNI < 55.5; Q4: PNI ≥ 55.5.

### 2.4 Primary outcomes

The outcomes included cardiovascular mortality and all-cause mortality. Mortality data were obtained from the NHANES Public-Use Linked Mortality File, which was linked to the National Death Index (NDI) and included follow-up data from participant enrollment until 31 December 2019. All-cause mortality was defined as death from any cause during the follow-up period, as determined by the cause-of-death codes provided by the NDI. Cardiovascular mortality was defined as death attributable to CVD, including coronary artery disease, heart failure, arrhythmias, stroke, and others. These events were identified using the International Classification of Diseases, Tenth Revision (ICD-10) codes recorded in the NDI, specifically codes within the range of I00–I99. Follow-up time was calculated as the duration (in months) from the baseline survey to the date of death or the end of follow-up (31 December 2019). For participants who did not experience a mortality event, their data were censored at the end of the follow-up period.

### 2.5 Variable assessment

This study systematically evaluated a range of variables, including demographic characteristics, clinical parameters, laboratory indicators, and other relevant covariates. All data were derived from standardized measurements and questionnaires in the NHANES database. Demographic data included age, sex, race/ethnicity, marital status, and education level. Race/ethnicity was categorized as Mexican American, Hispanic, non-Hispanic White, non-Hispanic Black, and other races. Education level was classified as “Less than high school,” “High school or equivalent,” and “College or above.” The poverty-income ratio (PIR) was defined as the ratio of family income to the poverty threshold. Anthropometric measures included body mass index (BMI). Comorbidities such as hypertension, diabetes, and CVD were obtained through self-reported medical history. Substance use status was ascertained through self-reported questionnaires and categorized as follows: (1) Smoking status: never-smokers (< 100 cigarettes in lifetime) and smokers (≥ 100 cigarettes in lifetime); (2) Drinking status: drinkers (≥ 4 standard drinks/day; 1 drink = 14 g alcohol) and non-drinkers (< 4 drinks/day). Laboratory indicators were collected at mobile examination centers (MECs) by professionally certified and extensively trained medical technicians and phlebotomists.

### 2.6 Statistical analysis

All statistical analyses were performed using R software (version 4.3.3), and GraphPad Prism 9 was used for generating forest plots. Based on the PNI values of CKD patients, the cohort was divided into quartiles (Q1–Q4) for descriptive statistical analysis. Demographic information, clinical characteristics (e.g., age, sex, ethnicity, smoking history, alcohol consumption), and laboratory parameters were summarized. Continuous variables were expressed as mean ± standard deviation (SD), while categorical variables were presented as frequencies and percentages. For normally distributed data, one-way analysis of variance (ANOVA) was used; otherwise, the Kruskal–Wallis test was applied. Kaplan–Meier survival curves were plotted according to PNI quartiles, and differences in survival between groups were compared using the log-rank test. Survival time was defined as the duration from enrollment to the occurrence of events, including cardiovascular death and all-cause death. To evaluate the independent predictive value of PNI for cardiovascular and all-cause mortality, Cox proportional hazards regression models were constructed, adjusting for potential confounding variables. Death events were set as the dependent variable, while PNI (calculated from serum albumin, with albumin excluded from the model), demographic characteristics, clinical parameters, and laboratory indicators were included as independent variables. Both univariate and multivariate analyses were performed, and results were reported as hazard ratios (HRs) with 95% confidence intervals (CIs). Restricted cubic splines (RCS) were employed to examine the potential non-linear relationship between PNI and mortality outcomes in both univariate and multivariate analyses. To ensure the robustness of the findings, sensitivity analyses were conducted, including stratified analyses by age, sex, and the presence of comorbidities (e.g., diabetes, hypertension). Mediation analysis was performed to assess the mediating effects of eGFR and age on the association between PNI and mortality outcomes. A two-sided *p*-value < 0.05 was considered statistically significant for all hypothesis tests.

### 2.7 Ethical approval and consent to participate

This study utilized a publicly available dataset (NHANES), which was collected in accordance with ethical standards, including obtaining informed consent from all participants. All methods were conducted in compliance with relevant guidelines and regulations.

## 3 Results

### 3.1 Baseline characteristics

A total of 4,528 CKD patients were included in this study, comprising 2,179 males (43.16%) and 2,349 females (56.84%). The median age was 61 years (interquartile range [IQR]: 45–74). Among the participants, 3,170 (63.15%) had hypertension, 1,710 (30.74%) had diabetes, and 353 (6.43%) had CVD. By the end of the follow-up period, 1,725 deaths (33.00%) were recorded, including 685 cardiovascular deaths (12.85%). Based on quartiles of the PNI, patients were stratified into four groups: Q1 (PNI < 49.0, *n* = 1,155, 25.51%), Q2 (49.0 ≤ PNI < 52.5, *n* = 1,269, 28.03%), Q3 (52.5 ≤ PNI < 55.5, *n* = 1,026, 22.66%), and Q4 (PNI ≥ 55.5, *n* = 1,078, 23.81%). Significant differences (*P* < 0.05) were observed among the four groups in terms of sex, age, race, marriage, drinking, body mass index (BMI), hypertension, diabetes, CVD, and laboratory parameters, including serum albumin, blood urea nitrogen (BUN), creatinine, uric acid, calcium, phosphorus, potassium, triglycerides, total cholesterol (TC), high-density lipoprotein cholesterol (HDL-C), and eGFR. Patients in the lower PNI quartiles (Q1 and Q2) were older, had higher BMI, BUN, uric acid, creatinine, potassium, and HDL-C levels, as well as higher rates of all-cause and cardiovascular mortality. Conversely, these patients exhibited lower levels of serum albumin, calcium, phosphorus, TC, triglycerides, hemoglobin, and eGFR. No significant differences (*P* > 0.05) were observed in the remaining parameters (Table 1).

**TABLE 1:** Baseline characteristics of patients with CKD according to quartiles of PNI in NHANES (1999–2018).

Variable	Total (*n* = 4,528)	Q1 (*n* = 1,155)	Q2 (*n* = 1,269)	Q3 (*n* = 1,026)	Q4 (*n* = 1,078)	*F*/χ^2^	*P*
**Sex, *n* (%)**						15.614	0.032
Male	2,179 (43.16)	578 (45.78)	590 (41.23)	470 (39.46)	541 (46.29)		
Female	2,349 (56.84)	577 (54.22)	679 (58.78)	556 (60.54)	537 (53.71)		
Age (years)	61 (45, 74)	68 (55, 78)	64 (49, 75)	60 (43, 72)	53 (38, 68)	180.915	< 0.001
**Marriage, *n* (%)**						42.540	< 0.001
No	664 (16.17)	125 (11.17)	185 (14.57)	145 (17.26)	209 (21.00)		
Yes	3,864 (83.84)	1,030 (88.83)	1,084 (85.44)	881 (82.74)	869 (79.01)		
**Race, *n* (%)**						73.054	< 0.001
Mexican American	751 (7.11)	169 (6.06)	185 (5.80)	194 (7.65)	203 (8.83)		
Other Hispanic	327 (5.76)	62 (3.73)	93 (4.70)	78 (7.37)	94 (7.07)		
Non-Hispanic White	2,156 (68.66)	579 (71.14)	629 (71.53)	482 (67.11)	466 (65.08)		
Non-Hispanic Black	1,027 (12.86)	302 (16.15)	292 (12.95)	202 (11.81)	231 (10.95)		
Other race	267 (5.61)	43 (2.92)	70 (5.02)	70 (6.06)	84 (8.07)		
**Education, *n* (%)**						11.253	0.305
Less than high school	1,578 (25.19)	413 (25.26)	444 (25.64)	332 (22.70)	389 (26.93)		
High school or equivalent	1,113 (25.98)	277 (27.89)	313 (26.35)	268 (26.82)	255 (23.26)		
College or above	1,837 (48.83)	465 (46.85)	512 (48.02)	426 (50.48)	434 (49.82)		
**Smoking, *n* (%)**						7.020	0.289
No	2,269 (49.92)	591 (50.37)	647 (52.20)	488 (46.75)	543 (50.11)		
Yes	2,259 (50.08)	564 (49.63)	622 (47.81)	538 (53.25)	535 (49.89)		
**Drinking, *n* (%)**						20.099	0.009
No	1,631 (33.88)	450 (38.54)	474 (35.46)	338 (30.31)	369 (31.62)		
Yes	2,897 (66.12)	705 (61.46)	795 (64.54)	688 (69.69)	709 (68.38)		
PIR	2.42 (1.27, 4.43)	2.33 (1.29, 4.10)	2.47 (1.32, 4.69)	2.51 (1.27, 4.55)	2.31 (1.24, 4.120)	5.053	0.173
BMI (kg/m^2^)	28.40 (24.60, 33.47)	28.99 (24.85, 35.00)	28.50 (24.85, 33.60)	28.29 (24.25, 33.00)	28.14 (24.08, 33.09)	8.774	0.036
**Hypertension, *n* (%)**						49.104	< 0.001
No	1,358 (36.85)	292 (29.58)	354 (34.23)	337 (40.08)	375 (42.69)		
Yes	3,170 (63.15)	863 (70.42)	915 (65.77)	689 (59.92)	703 (57.31)		
**Diabetes, *n* (%)**						17.740	0.012
No	2,818 (69.26)	666 (64.25)	792 (69.04)	661 (71.76)	699 (71.40)		
Yes	1,710 (30.74)	489 (35.75)	477 (30.96)	365 (28.24)	379 (28.60)		
**CVD, *n* (%)**						12.895	0.017
No	4,175 (93.57)	1,044 (91.67)	1,165 (92.83)	963 (94.64)	1,003 (94.93)		
Yes	353 (6.43)	111 (8.33)	104 (7.17)	63 (5.36)	75 (5.07)		
Albumin (g/L)	42.256 ± 0.086	38.818 ± 0.092	41.705 ± 0.081	43.137 ± 0.108	44.901 ± 0.127	1,433.410	< 0.001
Blood urea nitrogen (mmol/L)	5.40 (3.93, 7.14)	6.10 (4.28, 8.21)	5.71 (4.28, 7.50)	5.36 (3.93, 7.14)	5.00 (3.93, 6.43)	75.411	< 0.001
Calcium (mmol/L)	2.37 ± 0.003	2.32 ± 0.004	2.36 ± 0.003	2.38 ± 0.004	2.40 ± 0.004	334.672	< 0.001
TC (mmol/L)	5.14 ± 0.03	4.98 ± 0.05	5.05 ± 0.05	5.20 ± 0.05	5.34 ± 0.05	32.211	< 0.001
Phosphorus (mmol/L)	1.20 ± 0.005	1.20 ± 0.008	1.192 ± 0.009	1.20 ± 0.008	1.22 ± 0.008	4.732	0.031
Triglycerides (mmol/L)	1.52 (1.03, 2.30)	1.36 (0.98, 1.95)	1.46 (1.00, 2.24)	1.52 (1.01, 2.30)	1.80 (1.16, 2.76)	39.275	< 0.001
Uric acid (mmol/L)	350.80 ± 2.18	361.14 ± 3.89	354.09 ± 3.86	343.72 ± 4.60	345.20 ± 4.16	9.640	0.002
Creatinine (μmol/L)	88.40 (69.84, 106.96)	91.94 (72.49, 116.69)	88.40 (70.72, 114.04)	81.33 (63.65, 106.08)	79.60 (62.76, 97.24)	102.813	< 0.001
Sodium (mmol/L)	139.00 (138.00, 141.00)	139.00 (137.00, 141.00)	139.00 (138.00, 141.00)	139.00 (137.90, 141.00)	139.00 (138.00, 140.80)	3.529	0.321
Potassium (mmol/L)	4.08 ± 0.01	4.16 ± 0.02	4.09 ± 0.02	4.05 ± 0.02	4.03 ± 0.023	19.691	< 0.001
Hemoglobin (g/dL)	14.06 ± 0.04	13.47 ± 0.07	13.86 ± 0.06	14.16 ± 0.06	14.65 ± 0.07	202.741	< 0.001
HDL-C (mmol/L)	1.29 (1.06, 1.60)	1.29 (1.09, 1.66)	1.32 (1.09, 1.60)	1.29 (1.06, 1.66)	1.22 (1.00, 1.50)	18.337	< 0.001
UACR (mg/g)	41.71 (15.76, 93.10)	40.25 (12.89, 105.66)	38.62 (11.05, 85.71)	41.11 (17.42, 85.57)	46.35 (31.02, 94.18)	7.844	0.054
eGFR (ml/min/1.73 m^2^)	80.23 ± 0.74	70.56 ± 1.20	74.97 ± 1.09	83.62 ± 1.32	90.623 ± 1.38	159.037	< 0.001
**ALL-cause mortality, *n* (%)**						175.33	< 0.001
No	2,803 (67.00)	553 (51.78)	774 (65.68)	691 (70.82)	785 (77.63)		
Yes	1,725 (33.01)	602 (48.22)	495 (34.32)	335 (29.18)	293 (22.31)		
**CVD mortality, *n* (%)**						95.850	< 0.001
No	3,843 (87.15)	903 (78.83)	1,086 (87.68)	890 (88.28)	964 (92.59)		
Yes	685 (12.85)	252 (21.18)	183 (12.32)	136 (11.73)	114 (7.41)		

Normal distribution: Mean ± SE, *p*-values via weighted ANOVA; non-normal distribution: M (P25, P75), *P*-values via weighted Kruskal–Wallis test; Categorical: *n*, %, *P*-values from weighted chi-square. PNI, prognostic nutritional index; Q1: PNI < 49.0, Q2: 49.0 ≤ PNI < 52.5, Q3: 52.5 ≤ PNI < 55.5, Q4: PNI ≥ 55.5. PIR, poverty income ratio; BMI, body mass index; TC, total cholesterol; HDL-C, high-density lipoprotein cholesterol; UACR, urinary albumin-to-creatinine ratio; eGFR, estimated glomerular filtration.

### 3.2 Kaplan–Meier curves for PNI in predicting cardiovascular and all-cause mortality

**FIGURE 2:**
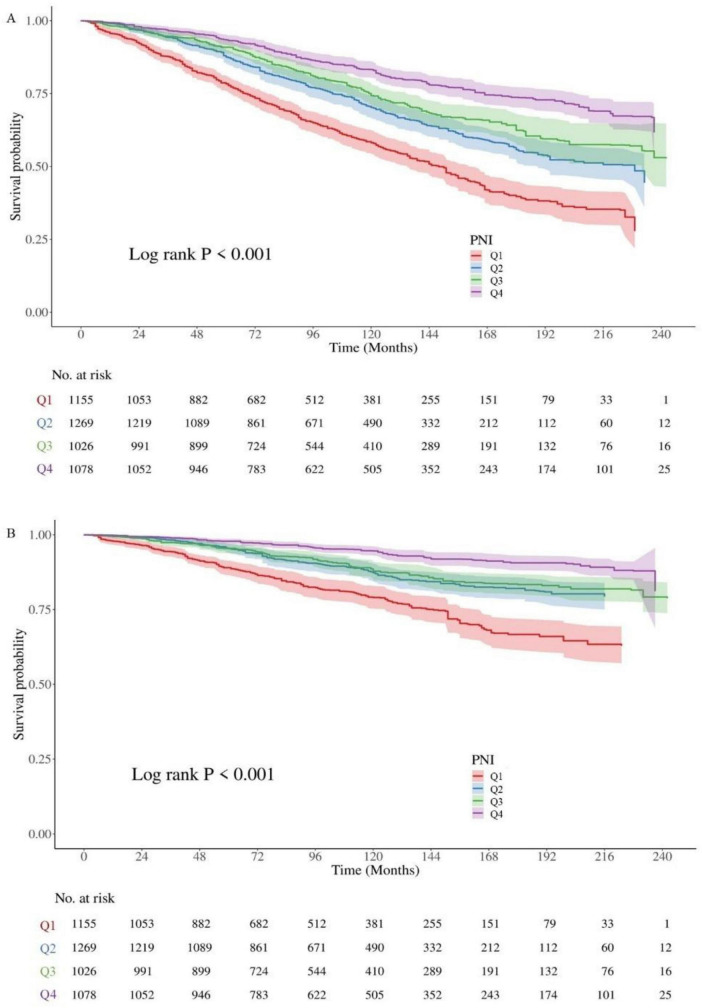
Kaplan–Meier curves for cardiovascular and all-cause mortality according to PNI levels. **(A)** Kaplan–Meier curve for all-cause mortality by PNI groups. **(B)** Kaplan–Meier curve for cardiovascular mortality by PNI groups. Log-rank tests confirmed significant differences between groups (*P* < 0.001). PNI: prognostic nutritional index; Q1: PNI < 49.0, Q2: 49.0 ≤ PNI < 52.5, Q3: 52.5 ≤ PNI < 55.5, Q4: PNI ≥ 55.5.

The results demonstrated that patients in the Q1 group (lowest PNI quartile) had significantly lower survival probabilities for both all-cause and cardiovascular mortality compared to those in the Q2, Q3, and Q4 groups (*P* < 0.001) (Figure 2).

### 3.3 Predictive value of PNI quartiles for cardiovascular and all-cause mortality risk

**FIGURE 3:**
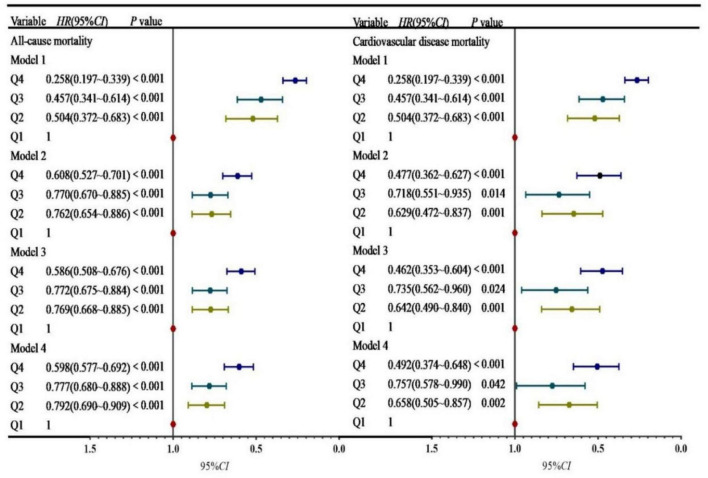
The predictive value of PNI groups for cardiovascular and all-cause mortality risk. Model 1: Unadjusted; Model. 2: Adjusted for confounders including age, sex, race, and marriage; Model 3: Adjusted for confounders including age, sex, race, marriage, hypertension, diabetes, CVD, and drinking; Model 4: Adjusted for age, sex, race, marriage, hypertension, diabetes, CVD, drinking, blood urea nitrogen, uric acid, creatinine, potassium, phosphorus, hemoglobin, and eGFR. Q1: PNI < 49.0, Q2: 49.0 ≤ PNI < 52.5, Q3: 52.5 ≤ PNI < 55.5, Q4: PNI ≥ 55.5.

Cox proportional hazards regression models were used to analyze the predictive value of PNI quartiles, with adjustments for potential confounders, including age, sex, ethnicity, marital status, alcohol consumption, hypertension, diabetes, CVD, and biochemical parameters. Using Q1 (lowest PNI quartile) as the reference group, the adjusted Cox regression analysis for all-cause mortality revealed significantly lower risks in Q2 (HR: 0.792, 95% CI: 0.690–0.909, *P* < 0.001), Q3 (HR: 0.777, 95% CI: 0.680–0.888, *P* < 0.001), and Q4 (HR: 0.598, 95% CI: 0.517–0.692, *P* < 0.001) compared to Q1. Similarly, in the Cox regression model for cardiovascular mortality, significantly reduced risks were observed in Q2 (HR: 0.658, 95% CI: 0.505–0.857, *P* = 0.002), Q3 (HR: 0.757, 95% CI: 0.578–0.990, *P* = 0.042), and Q4 (HR: 0.492, 95% CI: 0.374–0.648, *P* < 0.001) compared to Q1 (Figure 3).

### 3.4 Non-linear relationship between PNI and cardiovascular and all-cause mortality risk using RCS in cox regression models

**FIGURE 4:**
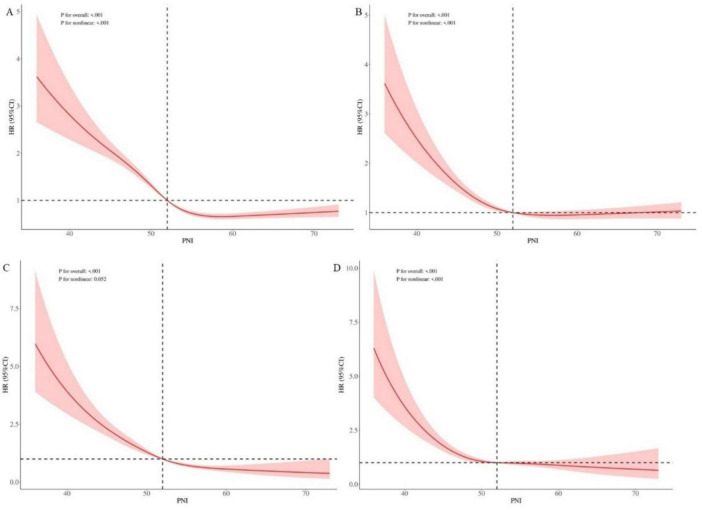
Restricted cubic spline regression analysis. **(A)** Non-linear relationship between PNI and all-cause mortality. **(B)** Non-linear relationship between PNI and all-cause mortality, adjusted for confounders. **(C)** Linear relationship between PNI and cardiovascular mortality. **(D)** Non-linear relationship between PNI and cardiovascular mortality, adjusted for confounders. **(B,D)** Adjusted for age, sex, race, marriage, hypertension, diabetes, CVD, drinking, blood urea nitrogen, uric acid, creatinine, potassium, phosphorus, hemoglobin, and eGFR. Knots were placed at 5, 27.5, 50, 72.5 and 95% of PNI distribution. The dashed vertical line indicates the 50th percentile knot (PNI = 52), which served as the reference point for non-linearity testing. PNI, prognostic nutritional index.

RCS within Cox regression models were employed to analyze the non-linear relationship between the PNI and the risks of cardiovascular and all-cause mortality. The results demonstrated a significant non-linear association between PNI and all-cause mortality risk in both unadjusted and adjusted models. When PNI values were below 52, the risk of mortality increased sharply with decreasing PNI levels. In contrast, when PNI values exceeded 52, the risk of mortality remained low and stabilized with increasing PNI levels. For cardiovascular mortality, the unadjusted RCS analysis revealed a significant linear relationship (*P* < 0.001), with mortality risk increasing significantly as PNI levels decreased. However, after adjusting for confounders, the RCS analysis indicated a significant non-linear relationship (*P* < 0.001). Similar to the all-cause mortality findings, when PNI values were below 52, the risk of cardiovascular mortality increased markedly with decreasing PNI levels, whereas PNI values above 52 were associated with lower and stable mortality risks (Figure 4).

### 3.5 Subgroup and interaction analyses of PNI and mortality outcomes

The results demonstrated that PNI significantly predicted all-cause mortality across subgroups stratified by sex, age, BMI, hypertension, and diabetes (*P* < 0.05). In the CVD subgroup, PNI showed significant predictive value for all-cause mortality in patients without CVD (HR: 0.94, 95% CI: 0.93–0.96, *P* < 0.001), but no significant association was observed in patients with CVD (HR: 0.96, 95% CI: 0.92–1.00, *P* = 0.079). In terms of CKD stages, PNI significantly predicted all-cause mortality in CKD stages 1, 4, and 5 (*P* < 0.05), but no significant association was found in CKD stages 2 and 3 (*P* > 0.05). For cardiovascular mortality, PNI demonstrated significant predictive value across all subgroups stratified by sex, age, BMI, hypertension, diabetes, and CVD (*P* < 0.05). In CKD stages, PNI significantly predicted cardiovascular mortality in stages 1, 2, 4, and 5 (*P* < 0.05), but no significant association was observed in stage 3 (*P* > 0.05). Interaction analyses revealed that sex (*P* = 0.036), age (*P* = 0.005), and CKD stage (*P* = 0.013) significantly modified the association between PNI and all-cause mortality. For cardiovascular mortality, age (*P* = 0.001) was identified as a significant effect modifier (Table 2).

**TABLE 2:** Subgroup analysis and interaction analysis.

Variables	*n* (%)	ALL-cause mortality			CVD mortality		
		HR (95% CI)	*P*-value	*P* for interaction	HR (95% CI)	*P*-value	*P* for interaction
All patients	4,528 (100.00)	0.95 (0.93∼0.96)	< 0.001		0.95 (0.93∼0.96)	< 0.001	
Sex, *n* (%)				0.036			0.15
Male	2,179 (48.12)	0.94 (0.92∼0.96)	< 0.001		0.91 (0.88∼0.94)	< 0.001	
Female	2,349 (51.88)	0.96 (0.93∼0.99)	0.003		0.93 (0.89∼0.97)	< 0.001	
Age (years)				0.005			0.001
< 60	1,604 (35.42)	0.90 (0.87∼0.94)	< 0.001		0.86 (0.82∼0.91)	< 0.001	
≥ 60	2,924 (64.58)	0.98 (0.96∼1.00)	0.015		0.95 (0.93∼0.97)	< 0.001	
BMI (kg/m^2^)				0.920			0.753
< 25	1,162 (25.66)	0.96 (0.93∼0.99)	0.022		0.93 (0.89∼0.96)	< 0.001	
25 ≤ and < 30	1,446 (31.93)	0.95 (0.92∼0.97)	< 0.001		0.92 (0.89∼0.95)	< 0.001	
≥ 30	1,920 (42.40)	0.94 (0.91∼0.97)	< 0.001		0.91 (0.88∼0.95)	< 0.001	
CVD, *n* (%)				0.077			0.135
No	4,175 (92.20)	0.94 (0.93∼0.96)	< 0.001		0.91 (0.89∼0.94)	< 0.001	
Yes	353 (7.80)	0.96 (0.92∼1.00)	0.079		0.95 (0.90∼1.00)	0.038	
Hypertension, *n* (%)				0.160			0.053
No	1,358 (29.99)	0.94 (0.90∼0.99)	0.015		0.91 (0.86∼0.96)	< 0.001	
Yes	3,170 (70.01)	0.95 (0.93∼0.97)	< 0.001		0.93 (0.90∼0.95)	< 0.001	
Diabetes, *n* (%)				0.109			0.092
No	2,818 (62.23)	0.94 (0.92∼0.96)	< 0.001		0.90 (0.88∼0.93)	< 0.001	
Yes	1,710 (37.77)	0.96 (0.93∼0.99)	0.003		0.93 (0.89∼0.97)	< 0.001	
CKD stage, *n* (%)				0.013			0.093
Stage 1	55 (1.21)	0.90 (0.81∼0.99)	0.039		0.86 (0.76∼0.97)	0.018	
Stage 2	132 (2.92)	1.00 (0.98∼1.02)	0.885		0.95 (0.88∼1.03)	0.202	
Stage 3	1,547 (34.17)	0.98 (0.95∼1.01)	0.197		0.96 (0.93∼0.99)	0.010	
Stage 4	1,259 (27.80)	0.94 (0.91∼0.97)	< 0.001		0.91 (0.88∼0.95)	< 0.001	
Stage 5	1,535 (33.90)	0.91 (0.88∼0.94)	< 0.001		0.88 (0.84∼0.93)	< 0.001	

BMI, body mass index; CKD, chronic kidney disease; CKD stage: Stage 1: eGFR ≥ 90, Stage 2: 60 ≤ eGFR < 90, Stage 3: 30 ≤ eGFR < 60, Stage 4: 15 ≤ eGFR < 30, Stage 5: eGFR < 15. The logistic regression model was used to estimate HR and 95% CI. Adjusted for confounders including race, marriage, drinking, blood urea nitrogen, uric acid, creatinine, potassium, phosphorus and hemoglobin.

### 3.6 Mediating effects of eGFR and age on the association between PNI and mortality outcomes

The mediation analysis revealed that in the association between PNI and all-cause mortality, the direct effect was significant (β = 1.07, 95% CI: 0.13–2.17, *P* < 0.001), while the indirect effect mediated by eGFR was not significant (β = −0.03, 95% CI: −0.10–0.01, *P* = 0.28). Similarly, for cardiovascular mortality, the direct effect of PNI was significant (β = 3.76, 95% CI: 2.61–4.92, *P* < 0.001), but the indirect effect mediated by eGFR was not significant (β = −0.01, 95% CI: −0.07–0.01, *P* = 0.48).

**FIGURE 5:**
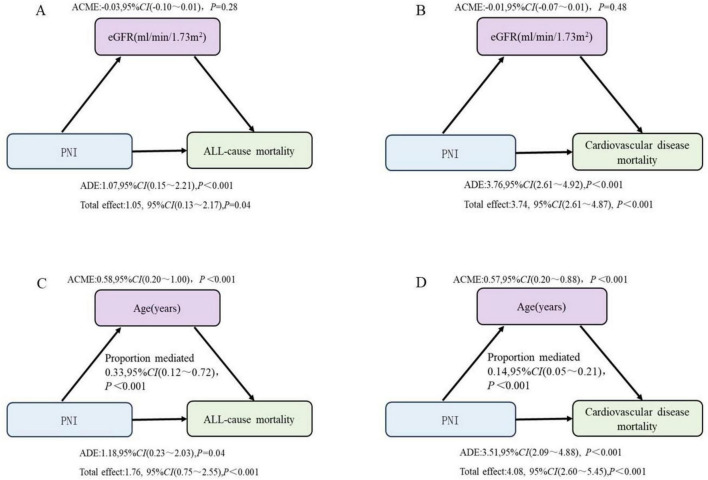
The mediating effects of eGFR and age on the relationship between PNI and mortality outcomes. **(A,B)** Adjusted for age, sex, race, marriage, hypertension, diabetes, CVD, drinking, blood urea nitrogen, uric acid, creatinine, potassium, phosphorus and hemoglobin. **(C,D)** Adjusted for sex, race, marriage, hypertension, diabetes, CVD, drinking, blood urea nitrogen, uric acid, creatinine, potassium, phosphorus, hemoglobin, and eGFR. PNI, prognostic nutritional index; eGFR, estimated glomerular filtration.

In contrast, age played a significant mediating role in the association between PNI and mortality outcomes. For all-cause mortality, both the direct effect (β = 1.18, 95% CI: 0.23–2.03, *P* = 0.04) and the indirect effect mediated by age (β = 0.58, 95% CI: 0.20–1.00, *P* < 0.01) were significant, with the indirect effect accounting for 33% of the total effect. For cardiovascular mortality, both the direct effect (β = 3.51, 95% CI: 2.09–4.88, *P* < 0.001) and the indirect effect mediated by age (β = 0.57, 95% CI: 0.20–0.88, *P* < 0.01) were significant, with the indirect effect contributing 14% of the total effect (Figure 5).

## 4 Discussion

CKD is one of the most prevalent and common diseases worldwide (19, 20). The PNI, a composite biomarker derived from serum albumin levels and peripheral blood lymphocyte counts, has gained widespread recognition for its prognostic value in various clinical conditions (21–23). PNI reflects both nutritional status and immune function, making it particularly relevant in chronic diseases. Studies have demonstrated a strong association between PNI and clinical outcomes, including all-cause mortality and cardiovascular mortality (24, 25). In patients with CVD, lower PNI values are significantly correlated with higher mortality rates and increased incidence of cardiovascular events (26). Specifically, PNI serves as an independent predictor of cardiovascular mortality, aiding in the identification of high-risk patients and providing early clues for intervention (27) CKD patients often experience severe malnutrition, with impaired immune function and poor nutritional status being key contributors to adverse outcomes (28, 29). This dual burden of immune decline and malnutrition not only exacerbates the clinical progression of CKD but also increases the risk of complications (30–32). The decline in PNI values, a composite marker of nutritional and immune status, reflects this clinical profile and further underscores its utility as a prognostic indicator in CKD patients. The findings of this study further support the prognostic value of PNI in CKD patients, demonstrating a significant association between low PNI and both all-cause and cardiovascular mortality.

This study revealed that patients in the low PNI group exhibited a distinct pattern of metabolic and immune dysregulation. These findings align closely with the pathophysiological features of CKD progression (33, 34), where low PNI patients commonly experience protein-energy wasting (PEW), chronic inflammation, and deteriorating renal function. Notably, despite the higher BMI observed in the low PNI group, this phenomenon is likely driven by metabolic syndrome-related fluid retention or abnormal fat distribution rather than indicating a favorable nutritional status (35, 36). Additionally, the elevated HDL-C levels in the low PNI group may be attributed to dysregulated hepatic lipoprotein synthesis in the context of chronic inflammation (37). A similar paradoxical observation was reported by Kon et al. (38) in a CKD cohort, underscoring the need for cautious interpretation of traditional lipid metrics in CKD patients.

The findings of this study demonstrate that patients in the low PNI group exhibited significantly lower survival probabilities for both all-cause and cardiovascular mortality compared to other groups. Previous studies, such as those by Barutcu Atas et al. (10) and Tsuda et al. (26), have also demonstrated that low PNI values are strongly associated with poor survival outcomes and increased mortality risk, particularly in patients with CVD and other chronic conditions, further validating the predictive value of PNI. Multivariate Cox regression analysis demonstrated an inverse association between PNI levels and mortality risk. Compared to patients with PNI < 49.0, those with PNI ≥ 55.5 exhibited lower risks of all-cause mortality (40% reduction) and cardiovascular mortality (51% reduction). These results are consistent with the findings of Chen et al. (39) and Hung et al. (40), indicating that patients with higher PNI values exhibit significant survival advantages when facing mortality risks. Additionally, the results of restricted cubic spline (RCS) analysis demonstrated a significant non-linear association between PNI and all-cause mortality risk, with a particularly pronounced increase in mortality risk when PNI values fell below 52. In contrast, mortality risk stabilized when PNI values exceeded 52. This non-linear relationship aligns with the findings of Yu et al. (41), suggesting that PNI has a more substantial impact on mortality risk at lower levels. The RCS analysis provides a more nuanced quantitative model of the relationship between PNI and mortality outcomes, further highlighting the potential of PNI in clinical risk assessment. The predictive value of PNI is further underscored by its strong association with mortality risk.

Our study reveals the predictive value of the PNI across different subgroups. The results demonstrate that PNI significantly predicts both all-cause mortality and cardiovascular mortality in subgroups stratified by sex, age, BMI, hypertension, diabetes, and CVD, further supporting its potential as a widely applicable prognostic indicator across diverse populations. Notably, the predictive ability of PNI for cardiovascular mortality was particularly pronounced in patients with CKD stages 1, 4, and 5, whereas its predictive power was relatively weaker in patients with CKD stages 2 and 3. This differential performance aligns with broader challenges in CKD risk stratification, where biomarker utility often varies by disease severity. As Khandpur et al. (42) highlighted, the heterogeneity in disease progression and biomarker relevance across CKD stages necessitates stage-specific predictive models. The enhanced predictive value of PNI in advanced CKD may stem from its sensitivity to the malnutrition-inflammation complex syndrome (MICS), a key contributor to morbidity and mortality in late-stage CKD. Since MICS becomes increasingly prevalent as renal function declines, PNI’s ability to capture this pathophysiological mechanism likely explains its stronger association with adverse outcomes in stages 4–5. Conversely, the relatively weaker predictive performance in stages 2–3 suggests that alternative risk factors may dominate disease progression during these intermediate phases. Recent real-world evidence indicates that SGLT2 inhibitors demonstrate significant cardiovascular protective effects in advanced CKD patients, though their efficacy may vary by renal function stage (43). This aligns with the stage-specific predictive performance of PNI observed in our study, collectively suggesting that CKD pathophysiology including inflammation, metabolic dysregulation may evolve dynamically with disease progression. Future studies should investigate synergistic strategies combining nutritional interventions such as PNI-guided therapy with targeted pharmacotherapies such as SGLT2 inhibitors (44). Furthermore, mediation analysis uncovered a potential mechanism by which PNI influences mortality outcomes through age. In the context of all-cause mortality, age served as a significant mediator, accounting for 33% of the total effect. This result implies that age may exacerbate the impact of PNI on mortality outcomes by influencing immune and metabolic functions. Similarly, the mediating effect of age was also significant for cardiovascular mortality. However, eGFR did not exhibit a significant indirect effect as a mediator in the relationship between PNI and all-cause or cardiovascular mortality, suggesting that the predictive value of PNI for mortality outcomes in CKD patients may be independent of eGFR.

Recent studies have shown that microbial dysregulation was involved in CKD and its complications (45–47). Extensive publications have indicated that gut microbiota interacted with kidneys and played critical roles in the pathogenesis of disease (46). The latest publications indicated the change in *Lactobacillus* level in feces of CKD patients and rats (48–52). The observed association between low PNI may be partially associated with gut microbiota dysbiosis, particularly the depletion of beneficial *Lactobacillus* species, which are known to maintain intestinal barrier integrity and reduce systemic inflammation. Emerging evidence suggests that *Lactobacillus johnsonii* supplementation may mitigate CKD progression by modulating tryptophan metabolism (53). Our mediation analysis further supports this link, showing that systemic inflammation (as indicated by PNI), but not eGFR, independently predicts mortality. These findings underscore the primacy of immune-metabolic dysregulation over purely renal functional decline in driving adverse outcomes. Increasing studies have demonstrated that targeted probiotic interventions to restore beneficial microbial taxa such as *Lactobacillus* spp. could represent a novel strategy to ameliorate PNI and improve clinical outcomes in CKD (4, 54–56).

This study has several limitations that should be acknowledged. First, the cross-sectional design of NHANES, although useful for identifying associations between PNI and outcomes, precludes the establishment of causal relationships or temporal sequences and should be interpreted with caution. Additionally, the lack of long-term follow-up data may underestimate the impact of dynamic changes in nutritional status on prognosis. Future prospective cohort studies incorporating serial PNI measurements would help establish temporal relationships and causal pathways. Second, despite adjusting for major confounders through multivariate models, unrecorded clinical interventions (e.g., dialysis regimens, nutritional support) and acute events (e.g., infections, surgeries) may introduce residual confounding. These factors could independently influence both PNI and mortality, potentially attenuating or amplifying the observed associations. Subsequent studies should prioritize comprehensive clinical data capture, potentially through electronic health record linkage, to better account for these confounding factors. The application of advanced analytical methods, such as marginal structural modeling, may further help address time-varying confounding. Third, certain variables in NHANES, such as lifestyle and dietary data, rely on self-reporting, which may be subject to recall bias or social desirability bias. Furthermore, incomplete physiological measurements, particularly in elderly or critically ill patients, may affect data reliability. Future investigations would benefit from incorporating objective biomarkers of nutritional status and standardized clinical assessments to supplement self-reported measures (57–59). Moreover, the study population is based on a multiethnic cohort in the United States, and extrapolating the findings to other healthcare systems or racial groups requires further validation (60–62). This highlights the need for multinational validation studies examining PNI’s predictive performance across diverse populations with varying CKD management approaches and genetic backgrounds (63). While these limitations necessitate cautious interpretation of our findings, they also delineate clear pathways for future research. We particularly recommend: (1) longitudinal studies with repeated PNI assessments to establish temporal relationships; (2) intervention studies examining whether PNI-guided management improves outcomes; (3) development of standardized protocols for PNI measurement and interpretation across diverse clinical settings; and (4) multinational collaborations to validate PNI’s utility in different healthcare contexts. Such efforts would substantially strengthen the evidence base for PNI’s clinical application in CKD management.

## 5 Conclusion

Our study demonstrates that the prognostic nutritional index (PNI) serves as an independent predictor of both all-cause and cardiovascular mortality in CKD patients, with a clinically significant threshold identified at PNI < 52. The partial mediation by age suggests an important interaction between nutritional status, immune competence, and biological aging in CKD pathophysiology. These findings support the potential clinical utility of PNI for risk stratification in CKD management.

## Data Availability

The datasets presented in this article are not readily available because the data analysis for this study was conducted using the Zstats platform, a proprietary software tool. Due to licensing restrictions and the proprietary nature of the platform, we are unable to share the raw data directly. Requests to access the datasets should be directed to weiweisdfey@163.com.
